# Prediction of Protein Domain with mRMR Feature Selection and Analysis

**DOI:** 10.1371/journal.pone.0039308

**Published:** 2012-06-15

**Authors:** Bi-Qing Li, Le-Le Hu, Lei Chen, Kai-Yan Feng, Yu-Dong Cai, Kuo-Chen Chou

**Affiliations:** 1 Institute of Systems Biology, Shanghai University, Shanghai, China; 2 Key Laboratory of Systems Biology, Shanghai Institutes for Biological Sciences, Chinese Academy of Sciences, Shanghai, China; 3 Shanghai Center for Bioinformation Technology, Shanghai, China; 4 College of Information Engineering, Shanghai Maritime University, Shanghai, China; 5 Gordon Life Science Institute, San Diego, California, United States of America; Uni. of South Florida, United States of America

## Abstract

The domains are the structural and functional units of proteins. With the avalanche of protein sequences generated in the postgenomic age, it is highly desired to develop effective methods for predicting the protein domains according to the sequences information alone, so as to facilitate the structure prediction of proteins and speed up their functional annotation. However, although many efforts have been made in this regard, prediction of protein domains from the sequence information still remains a challenging and elusive problem. Here, a new method was developed by combing the techniques of RF (random forest), mRMR (maximum relevance minimum redundancy), and IFS (incremental feature selection), as well as by incorporating the features of physicochemical and biochemical properties, sequence conservation, residual disorder, secondary structure, and solvent accessibility. The overall success rate achieved by the new method on an independent dataset was around 73%, which was about 28–40% higher than those by the existing method on the same benchmark dataset. Furthermore, it was revealed by an in-depth analysis that the features of evolution, codon diversity, electrostatic charge, and disorder played more important roles than the others in predicting protein domains, quite consistent with experimental observations. It is anticipated that the new method may become a high-throughput tool in annotating protein domains, or may, at the very least, play a complementary role to the existing domain prediction methods, and that the findings about the key features with high impacts to the domain prediction might provide useful insights or clues for further experimental investigations in this area. Finally, it has not escaped our notice that the current approach can also be utilized to study protein signal peptides, B-cell epitopes, HIV protease cleavage sites, among many other important topics in protein science and biomedicine.

## Introduction

Protein domains are structural, evolutionary and functional units of proteins. Prediction of protein domains from the sequence information can facilitate the prediction of protein tertiary structure [1], [2], the annotation of protein functions [2], [3], the protein structure determination [4], protein engineering [5] as well as mutagenesis [6], [7]. Particularly, the functional domains are actually the cores of proteins that play the major role for their functions. That is why in determining the 3D (three dimensional) structure of a protein by experiments (see, e.g., [8], [9], [10], [11]) or by computational modeling (see, e.g., [7], [12], [13], [14], [15]) the first priority was always focused on its functional domain. This is because the knowledge of protein functional domains is important for both basic research and drug development. Recently, the functional domain information of proteins has been widely used to formulate protein samples through the conception of pseudo amino acid composition [16], [17] for predicting various important attributes of proteins, such as membrane proteins and their types [18], GPCRs and their types [19], [20], proteases and their types [21], protein quaternary structural attribute [22], [23], protein structural classification [24], and protein subcellular localization [25], [26], [27]. Meanwhile, the protein domain information was also used to help analyzing protein-protein binding interactions [28], [29] and predicting the network of substrate-enzyme-product triads [30].

With the avalanche of protein sequences generated in the postgenomic age, many efforts have been made in hopes to predict the domains of proteins from their primary sequences alone. They can be roughly divided into three categories: (i) template-based method [31], [32], [33], (ii) ab-initio method [34], [35], and (iii) hybrid method by combining the aforementioned two [36], [37], [38]. Most template-based approaches attempted to find homologous sequences in the existing domain databases and then infer the domains of the query protein from these sequences. The obvious drawback of the template-based method was that it would work only when a domain was conserved and had already been deposited in a database. In other words, such an approach would fail to work if the query protein did not have significant sequence similarity to any of the domain-known proteins. In contrast to the template-based method, the ab-initio method could make predictions basically only based on the primary sequence information alone, and hence for those query proteins without significant sequence similarity to any of the domain-known proteins, the ab-initio method would be a good choice. The concreted techniques involved in the ab-initio methods are the machine learning algorithms [35], [39], artificial neural networks [40], and support vector machines [41], [42], along with the high quality domain databases such as CATH [43], SCOP [44] and DALI [45]. However, since it needed to scan the entire sequence of a protein usually involving several hundreds of amino acids, and also relied on the inputs containing weak domain information, the ab-initio method needed much more computational time and also often suffered from low prediction accuracy. The third method, or the hybrid method [36], [37], [46], took the strategy by integrating the template-based method and the ab-initio method. In the mean time, many softwares and web-server tools were developed for predicting protein domains, such as FIEFDom [47], DoMpro [40], DROP [42], DomCut [48] and Globplot [49]. Most of these tools aimed at predicting domain linker or domain boundary, and then followed by inferring the domain region.

The present study was initiated in an attempt to address the problem from such a keypoint by first identifying each of the constituent amino acid residue in a query protein belonging to the domain region or not. The techniques involved were RF (random forest), mRMR (maximum relevance minimum redundancy), and IFS (incremental feature selection). The amino acid features incorporated were the sequence conservation, residual disorder, secondary structure propensity, and solvent accessibility.

As summarized in a review [17] and demonstrated by a series of recent publications [50], [51], [52], [53], [54], [55], [56], to establish a really useful statistical predictor for a protein system, we need to consider the following procedures: (i) construct or select a valid benchmark dataset to train and test the predictor; (ii) formulate the protein samples with an effective mathematical expression that can truly reflect their intrinsic correlation with the target to be predicted; (iii) introduce or develop a powerful algorithm (or engine) to operate the prediction; (iv) properly perform cross-validation tests to objectively evaluate the anticipated accuracy of the predictor. Below, let us describe how to deal with these steps.

## Materials and Methods

### 1. Benchmark Dataset

A total of 517,100 protein sequences were retrieved from UniProt/Swiss-Prot database (version 2010_06) [57]. In order to construct a high-quality benchmark dataset, protein sequences were screened strictly according to the following criteria. (i) To reduce redundancy and homology bias, the cutoff threshold was set at 25% as suggested in [58], meaning that those sequence samples were removed by means of the program CD-HIT [59] that had 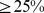 pairwise sequence identity to any other in the dataset. (ii) Of the remaining 45,942 protein samples obtained via the above winnowing procedure, only 9,409 were kept that had clear experimental domain annotations. (iii) Of the samples obtained via the above step, 110 proteins were removed because their disorder feature could not be calculated. Finally, a total of 9,299 protein sequences were obtained for the benchmark dataset 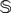 used in this study.

Furthermore, on the basis of the benchmark dataset 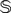, two working datasets, i.e., a learning (training) dataset 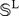 and an independent testing dataset 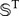, were constructed. In order to fully use the data in 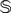 and meanwhile guarantee that 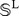 and 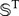 be completely independent of each other, the following condition was imposed:
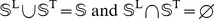
(1)where 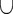, 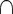, and 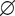 represent the symbols for “union”, “intersection”, and “empty set” in the set theory, respectively. Constrained by the condition of **Eq.1**, 8,000 protein sequences were randomly picked for the learning dataset 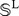 and the remaining 1,299 sequences for the testing dataset 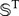. See the Online Supporting Information S1 for the codes of the proteins included in the two datasets, 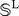 and 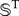, respectively.

Three different sliding windows [60] were used to generate the positive and negative datasets for this study: size-13, size-15, and size-17. For the size-13 window, we extracted all the 13-residue segments along a protein chain. The segments thus obtained can be denoted as 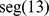 and classified into the following two groups:
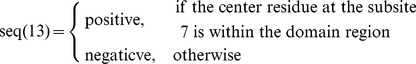
(2)


During the operation of sliding the window along a protein chain (cf. Figure 4 of [61], not all segments thus generated contain 13 amino acid residues. For those with less than 13 residues such as the ones generated at the positions close to the N-terminal or C-terminal, we complement their subsites with the nominal amino acid “X” to make them contain 13 residues as well. Thus, we obtained 1,694,782 positive samples and 4,093,531 negative samples from the learning dataset 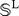. Subsequently, for each of the two sets of 13-residue samples, the program CD-HIT [59] was used to remove those that had 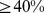 pairwise sequence identity to any other in a same set. Finally, we obtained 121,013 positive samples and 242,026 negative samples; i.e.,
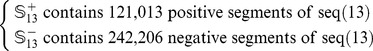
(3)where 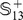 represents the positive learning dataset derived from 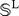 using the size-13 sliding window according to **Eq.2**, while 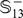 the corresponding negative dataset derived from 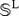.

By following the same procedure but using size-15 and size-17 sliding windows, respectively, we obtained
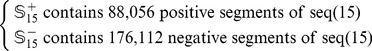
(4)and
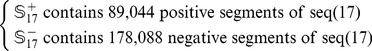
(5)


Now, the similar operation was made with the sliding windows on the 1,299 sequences in the testing dataset 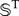, and we obtained 250,208 positive samples and 573,791 negative samples, respectively; i.e.,
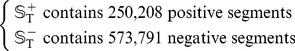
(6)where 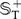 represents the positive learning dataset derived from 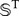, while 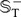 the corresponding negative dataset.

### 2. Feature Construction and Computational Method

#### 2.1 The features of PSSM conservation scores

Biology is a natural science with historic dimension. All biological species have developed starting out from a very limited number of ancestral species. The evolution in protein sequences involves changes of single residues, insertions and deletions of several residues [13], gene doubling, and gene fusion. In the course of time such changes accumulate, so that many similarities between initial and resultant amino acid sequences are eliminated, but the corresponding proteins may still share many common attributes, such as containing to a same domain and possessing basically the same function. In view of this, evolutionary conservation will play important roles in biological analysis: a more conserved residue within a protein sequence may indicate that it is more important for the protein function and thus under stronger selective pressure. To incorporate this kind of evolutionary effects, we used PSSM (position-specific scoring matrix) [62] generated by Position Specific Iterative BLAST (PSI BLAST) [63] to measure the conservation status for a specific residue. A 20-dimensional vector was used to denote the probabilities of conservation against mutations to 20 different amino acids for a specific residue. For a given sequence with 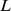, its PSSM would correspond to a 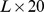 matrix, as formulated by equation 12 of [54]. Similar PSSM approaches have been successfully used to enhance the prediction quality for various protein attributes (see, e.g., [21], [26], [27], [50], [54], [55], [56], [64], [65], [66], [67], [68], [69].

#### 2.2 The features of amino acid factors

Since each of the 20 amino acids has specific but different properties, the composition of these properties of different residues within a protein may have impacts on its structure and function. AAIndex [70] is a database containing various physicochemical and biochemical properties of amino acids. Atchley et al. [71] performed multivariate statistical analyses on AAIndex and transformed AAIndex to five multidimensional and highly interpretable numeric patterns of attribute covariation that could reflect (i) polarity, (ii) secondary structure, (iii) molecular volume, (iv) codon diversity, and (v) electrostatic charge. Such five numerical pattern scores, denoted as AAFactor (amino acid factors), were used in this study to represent the respective properties of each amino acid in a given protein.

#### 2.3 The features of disorder score

Protein segments lacking fixed three-dimensional structures under physiological conditions play important roles in biological functions [72], [73], [74]. The disordered regions of proteins allow for more modification sites and interaction partners and always contain PTM (post translational modification) sites, sorting signals, and protein ligands. Therefore they are quite important for protein structure and function [72], [75], [76]. In this study, the program VSL2 [77], which can accurately predict both long and short disordered regions in proteins, was used to calculate the disorder score that denotes the disorder status of each amino acid in a given protein sequence.

#### 2.4 The features of secondary structure and solvent accessibility

As is well known, the function of a protein is closely correlated with its structure, and the post-translational modification of specific residues may be affected by the solvent accessibility of the relevant residues. Therefore, it would be useful during the process of encoding the constituent amino acids by also taking into account the features such as the secondary structure propensity and solvent accessibility. These kinds of features could be predicted by the software SSpro4 [78]. The second structural propensity predicted by SSpro4 for each amino acid was “helix”, “strand”, or “other”, encoded with “100”, “010” and “001”, respectively; the solvent accessibility as ‘buried’ or ‘exposed’, encoded with “10” and “01”, respectively.

#### 2.5 Feature space and feature vector

Each of the residues in a given protein segment was formulated in terms of 31 features, of which 20 from the PSSM scores, 1 from the disorder score, 5 from the AAFactor, 3 from the secondary structural propensities, and 2 from the solvent accessibility states. Thus, each of the segment samples generated by the size-13 sliding window would contain 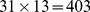 features; that by the size-15 sliding window, 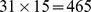; and that by the size-17 sliding window, 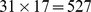. According to the general form of pseudo amino acid composition (cf. equation 6 of [17], each of these segments can be formulated by the following feature vector:
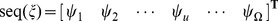
(7)where 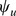 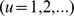 represents the 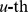 feature score, 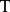 the transpose operator, and
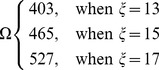
(8)For those segments that contain the nominal residue “X”, the corresponding subsite was substituted with zero.

#### 2.6 The mRMR method

In this study, the mRMR (minimal-redundancy-maximal-relevance) criterion [79] was used to rank the importance of the features. The mRMR method could rank the features according to their relevance to the target concerned and the redundancy among the features themselves. The ranked feature with a smaller index indicates that it has a better trade-off between the maximum relevance and minimum redundancy. To quantify both the relevance and redundancy, the following mutual information (MI) is defined to estimate how one vector is related to another:
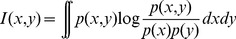
(9)where *x*, *y* are two vectors, 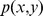 is the joint probabilistic density, *p(x)* and *p(y)* are the marginal probabilistic densities. Suppose *G* denotes the entire space containing all the feature components, *G*_s_ denotes the already-selected feature set containing *m* features, and *G*_t_ denotes the to-be-selected feature set containing *n* features. The relevance *D* between the feature *f* in *G* and the target *c* can be calculated by
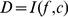
(10)The redundancy 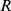 between the feature 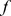 in *G*_t_ and all the features in *G*_s_ can be calculated by
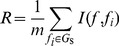
(11)To get the feature 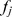 in *G*_t_ with the maximum relevance and minimum redundancy, let us combine **Eq.10** with **Eq.11**, as formulated by
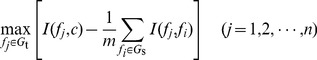
(12)The mRMR feature evaluation would continue 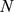 rounds when given a feature set with 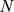 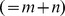 features. After these evaluations, a feature set 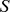 can be obtained by the mRMR method as formulated below
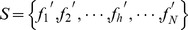
(13)where each feature in 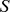 has a subscript index indicating at which round the feature is selected. The better the feature is, the earlier it has been selected.

The mRMR program can be downloaded from the web-site at http://penglab.janelia.org/proj/mRMR/.

#### 2.7 The RF (random forest) method

The RF approach is a popular machine-learning algorithm that has been recently successfully used in dealing with various biological prediction problems (see, e.g., [38], [52], [80], [81], [82], [83]). Developed by Loe Breiman [84], RF is an ensemble predictor consisting of multiple decision trees. In Weka 3.6.4 [85], the classifier named with “RandomForest” has implemented the predictor. In the current study, RandomForest was adopted as the prediction engine and operated with the default parameters. During the process of classifying a queried sample with its feature vector, RandomForest first grew 10 decision trees according to the following procedures. (i) Suppose the number of training cases is *N*, take *N* samples at random – but *with replacement*, from the original data. These samples are to form the training set for growing the tree. Here the so-called “with replacement” is a mathematical term meaning that a sample selected at random from the original dataset is returned to the original dataset before a second one is selected at random. In other words, whenever a sample is selected, the original dataset contains all the same samples. Thus, an exactly same sample may be selected more than once, and there is no change at all in the size of the original dataset at any stage. (ii) If each case consists of *M* input features, choose a number *m* = [log_2_ *M*+1] which is much less than *M*. At each node, *m* features are selected randomly out of the *M* features and the most optimized split on these *m* features is employed to split the node. The value of *m* does not change during the growth of the tree. (iii) Each tree is fully grown and not pruned. Then the input vector is predicted by each of 10 decision tree and 10 predicted classes provided by them are obtained. Finally, the class with the most votes will be selected as the output class of RandomForest.

The Weka program package can be downloaded from the web-site at http://www.cs.waikato.ac.nz/ml/weka/index_downloading.html

#### 2.8 The cross-validation method

In statistical prediction, the following three cross-validation methods are often used to examine a predictor for its effectiveness in practical application: independent dataset test, subsampling test, and jackknife test [86]. However, as elucidated in [58] and demonstrated by Eqs.28–32 of [17], among the three cross-validation methods, the jackknife test is deemed the least arbitrary (most objective) that can always yield a unique result for a given benchmark dataset, and hence has been increasingly used and widely recognized by investigators to examine the accuracy of various predictors (see, e.g., [20], [26], [27], [87], [88], [89], [90], [91]). However, to reduce the computational time, we adopted the 5-fold cross-validation in this study as done by many investigators with SVM as the prediction engine (see, e.g., [92], [93], [94]). During the process of 5-fold cross-validation, the benchmark dataset was first equally divided into 5 subsets. Subsequently, each of the subsets was in turn used as the testing dataset and the remaining four subsets as the training or learning dataset. To evaluate the performance of the predictor, the prediction accuracy, specificity, sensitivity and MCC (Matthews's correlation coefficient) were calculated below:
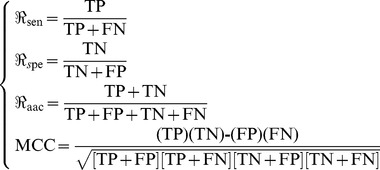
(14)where 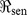 reflects the sensitivity, i.e., the rate of positive samples that are correctly predicted as positive; 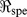 reflects the specificity, i.e., the rate of negative samples that are correctly predicted as negative; 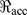 reflects the accuracy, i.e., the rate of correctly predicted events; MCC is the Matthew's correlation coefficient; TP represents the true positive; TN, the true negative; FP, the false positive; and FN, the false negative.

#### 2.9 The IFS (incremental feature selection) approach

Based on the ranked features according to their importance evaluated by the mRMR approach, we used the IFS [95], [96], [97] approach to determine the optimal number of features. During the IFS procedure, features in the ranked feature set were added with a stepwise of 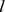 from higher to lower rank. A new feature set was formed when 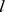 features had been added. Thus 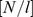 feature sets would be composed for *N* ranked features. The *i*-th feature set is:
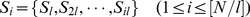
(15)where *N* denotes the total number of features in the original dataset and *l* (step) is a positive integer. In this study 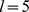. For each of the [N/*l*] feature sets, an RF classifier was constructed and examined using the 5-fold cross-validation on the benchmark dataset. By doing so we obtained an IFS table with one column for the index *i* and the other four columns for the prediction accuracy, sensitivity, specificity and MCC, respectively. Thus, we could obtain the optimal feature set (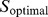), with which the predictor would yield the best prediction performance.

#### 2.10 The final optimal feature set

The MCC curve was fluctuating with the increase of feature numbers. Therefore, it was necessary to carefully examine its variation against the increasing feature number. In this study the feature-increasing gap was set at 5 to winnow out the optimal features. In other words, we compared two neighbor MCC values at a time with a stepwise of five features, if the latter MCC value is greater than the former one, then the corresponding five features were reserved to join the final optimal feature set; otherwise, discarded. The final optimal feature set thus established consisted of 195 features and would be used for further analysis.

We installed Weka into our Linux machine. Its “Run Environment and Configuration” was: Hardware 2 Intel(R) Xeon(R); CPU E5335@2.00 GHz; 16 G RAM; OS CentOS release 4.9 (Final) x86_64.

## Results and Discussion

### 1. The mRMR Result

Listed in the Online Supporting Information S2 are two kinds of outcomes obtained by running the mRMR software: one is called the “MaxRel feature list” that ranked all the features according to their relevance to the class of samples; the other one is the “mRMR feature list” that ranked the features according to the criteria of maximum relevance and minimum redundancy. In the mRMR feature list, the smaller the index of a feature was, the more important the feature would be for the protein domain prediction. Accordingly, the mRMR feature list could be used to establish the optimal feature set in the IFS procedure.

### 2. IFS and Final Optimal Feature Set

**Figure 1:**
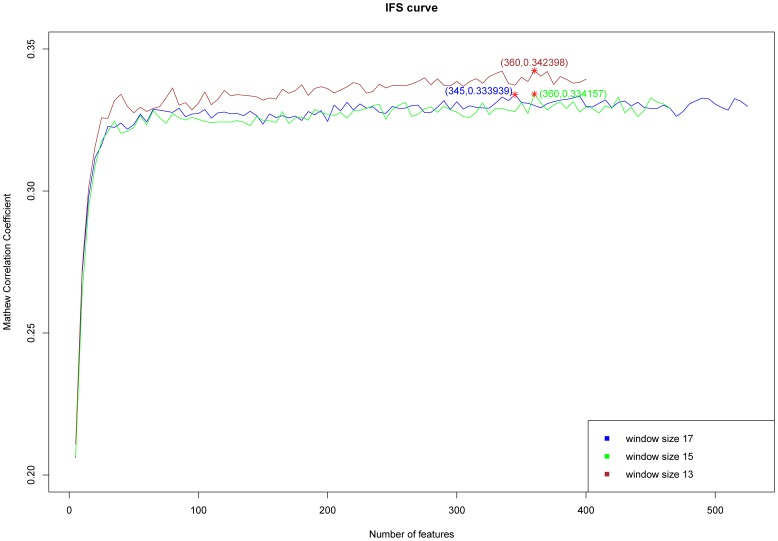
A plot to show the change of the MCC values versus the feature numbers with different window sizes. The IFS curves were drawn based on the data in Online Supporting Information S3. The MCC value reached the peak when the number of feature = 360 and the window size = 13. The 360 features thus obtained were used to form the optimal feature set for the protein domain predictor. Purple line is for the case of size-17 window, green for size-15 window, and brown for size-17 window. See the text for further explanation.

In Section 2.9 of Materials and Methods, by setting 403 for *N* and 5 for the feature-increasing gap, 80 individual predictors corresponding to 80 feature subsets were established for predicting the protein domain sites in the sequence samples generated by the size-13 sliding window. Listed in the Online Supporting Information S3 are the rates of prediction accuracy, specificity, sensitivity and MCC (cf. 14) obtained by each of the 80 predictors. Shown in **Fig. 1** is the IFS curve plotted based on the data in Online Supporting Information S3. The same calculations were also carried out for the size-15 and size-17 windows, and the corresponding results were also plotted in **Fig. 1**, from which we can see that the predictor based on the size-13 window outperformed the other two, and that the maximal MCC was 0.342 when 360 features were included. These 360 features were deemed to form the optimal feature set of our classifier. With such a classifier, the prediction sensitivity, specificity and accuracy were 0.577, 0.768 and 0.704 respectively (**Table 1**). The optimal 360 features were given in the Online Supporting Information S4. After taking the IFS procedure (cf. In Sections 2.9 and 2.10 of Materials and Methods), we obtained the 195 final optimal features as given in the Online Supporting Information S5.

**Table 1:** The predicted results obtained with different window size.

Window size	Dataset	Sensitivity^c^ 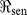	Specificity^c^ 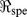	Accuracy^c^ 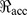	MCC^c^
13	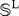 a	0.577	0.768	0.704	0.342
	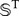 b	0.578	0.794	0.728	0.367
15	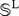 a	0.570	0.766	0.701	0.334
	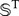 b	0.571	0.793	0.726	0.360
17	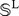 a	0.569	0.767	0.701	0.333
	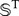 b	0.574	0.793	0.726	0.362

a 5-fold crossover test based on the learning dataset 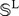 (cf. **Eq.1**).

b Using the rule trained by 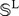 to predict the query proteins in the independent dataset 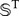 (cf. **Eq.1**).

c See Eq.14 for more explanation.

Hereafter, all the analyses will be based on such 195 final optimal features.

The CPU time of the above calculation for size-13, 15 and 17 windows were about 4 hours, 5 hours and 6 hours, respectively.

### 3. Feature Analysis

**Figure 2:**
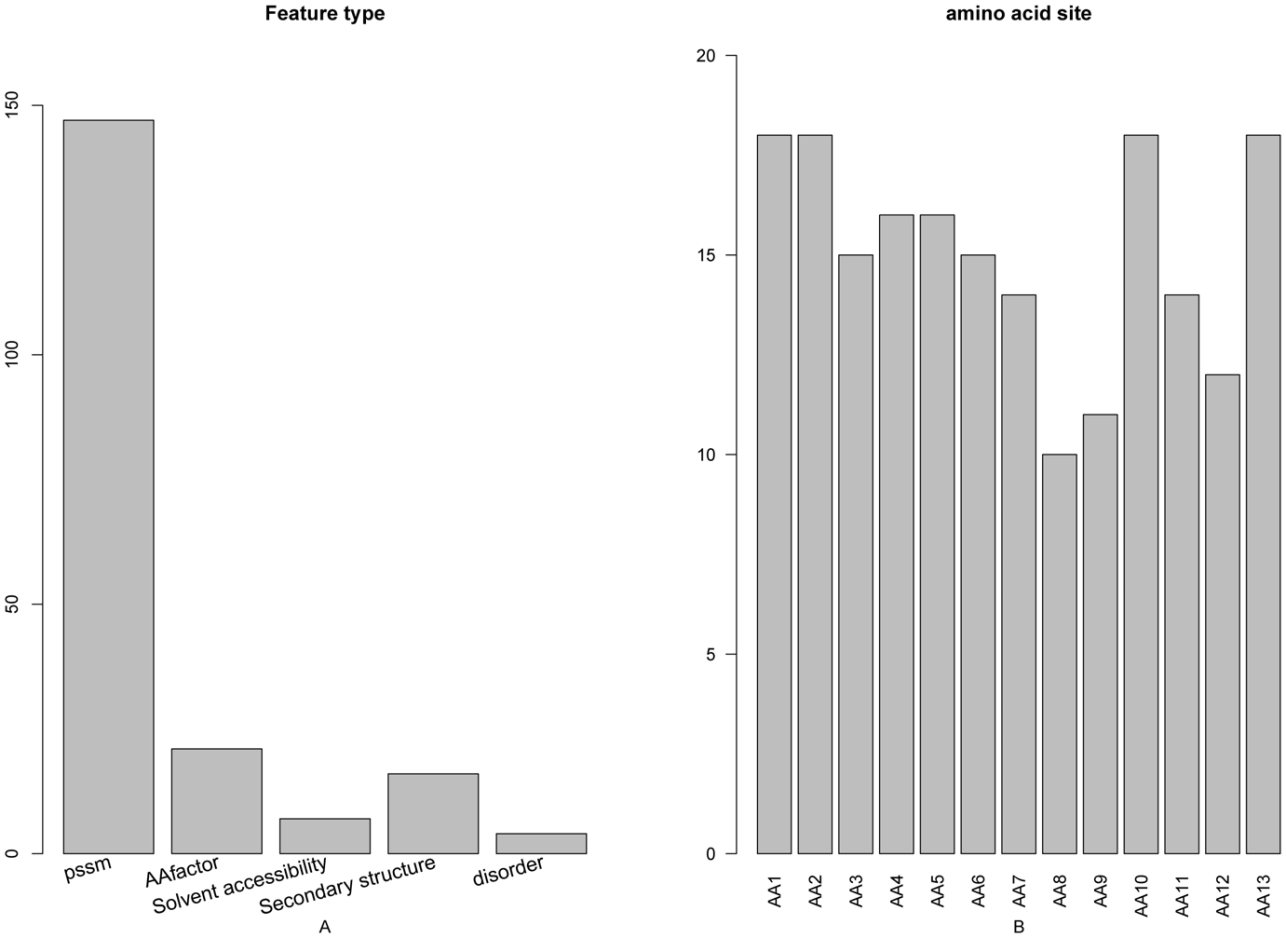
A 2-dimensional histogram to characterize the final optimal features set. The impact on the domain prediction from (**A**) the five different feature types, and (**B**) each of the 13 subsites. See the text for further explanation.

The distribution of the number of each type of features in the final optimal feature set was investigated and shown in **Fig. 2A**. Of the 195 optimal features, 147 were from PSSM conservation scores, 21 from the amino acid factors, 4 from the disorder scores, 7 from the solvent accessibilities, and 16 from the secondary structural propensities. All these five kinds of features made contributions to the prediction of protein domain sites. It was revealed by the site-specific distribution of the optimal feature set (see **Fig. 2B**) that sites 1–2, site 10 and site 13 played most important roles in determining the domain sites. In addition, the features of site 4 and site 5 also had considerable impacts on the prediction of protein domain sites.

### 4. PSSM Conservation Score Feature Analysis

**Figure 3:**
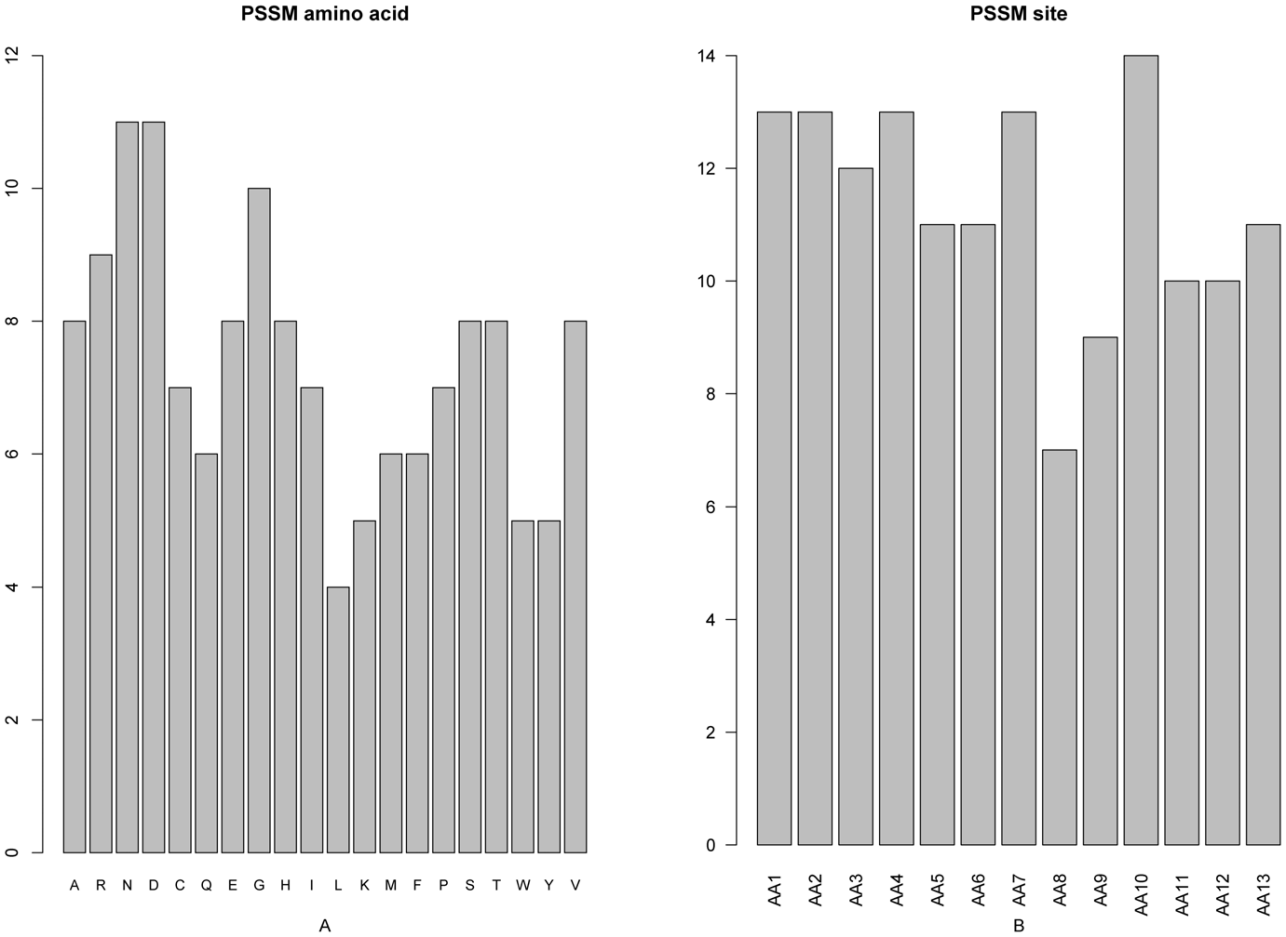
A 2-dimensional histogram to characterize the PSSM features in the final optimal features set. (**A**) The impact on the domain prediction from the mutation to each of the 20 amino acid types. (**B**) The evolutional conservation status for each of the 13 subsites. See the text for further explanation.

As mentioned above, among the 195 optimal features, 147 belonged to the PSSM conservation features and hence had the highest proportion. It can be clearly seen from **Fig. 3A** that each of the 20 different amino acid types would have different PSSM conservation impact in determining the protein domain site. In this regard, the amino acid N (asparagine) or D (aspartic acid) would have the highest impact, successively followed by G (glycine), R (arginine), and so forth. Interestingly, it has been reported that D, G and R were over-represented in protein interaction domains [98]. Besides, G was believed to be instrumental in defining the core domain and inter-domain regions of a protein [39]. Meanwhile, as shown in **Fig. 3B**, for the samples generated by the size-13 window (cf. **Eq.2**), the conservation status at the subsite 10 played the most important role in predicting the protein domain site, followed by the subsites 1, 2, 4, and 7. Furthermore, of the top ten features in the final optimal feature list, five were from the PSSM conservation features. The first one was the conservation status against residue M (methionine) at subsite 1 (index 3, “AA1_pssm_13”). The other four were the conservation status against residue A at subsite 12 (index 4, “AA12_pssm_1”), the conservation status against residue G at subsite 6 and site 4 (index 6 and index 7, “AA6_pssm_8” and AA4_pssm_8), and the conservation status against residue T (threonine) at subsite 2 (index 8, “AA2_pssm_17”)

### 5. Amino Acid Factor Analysis

**Figure 4:**
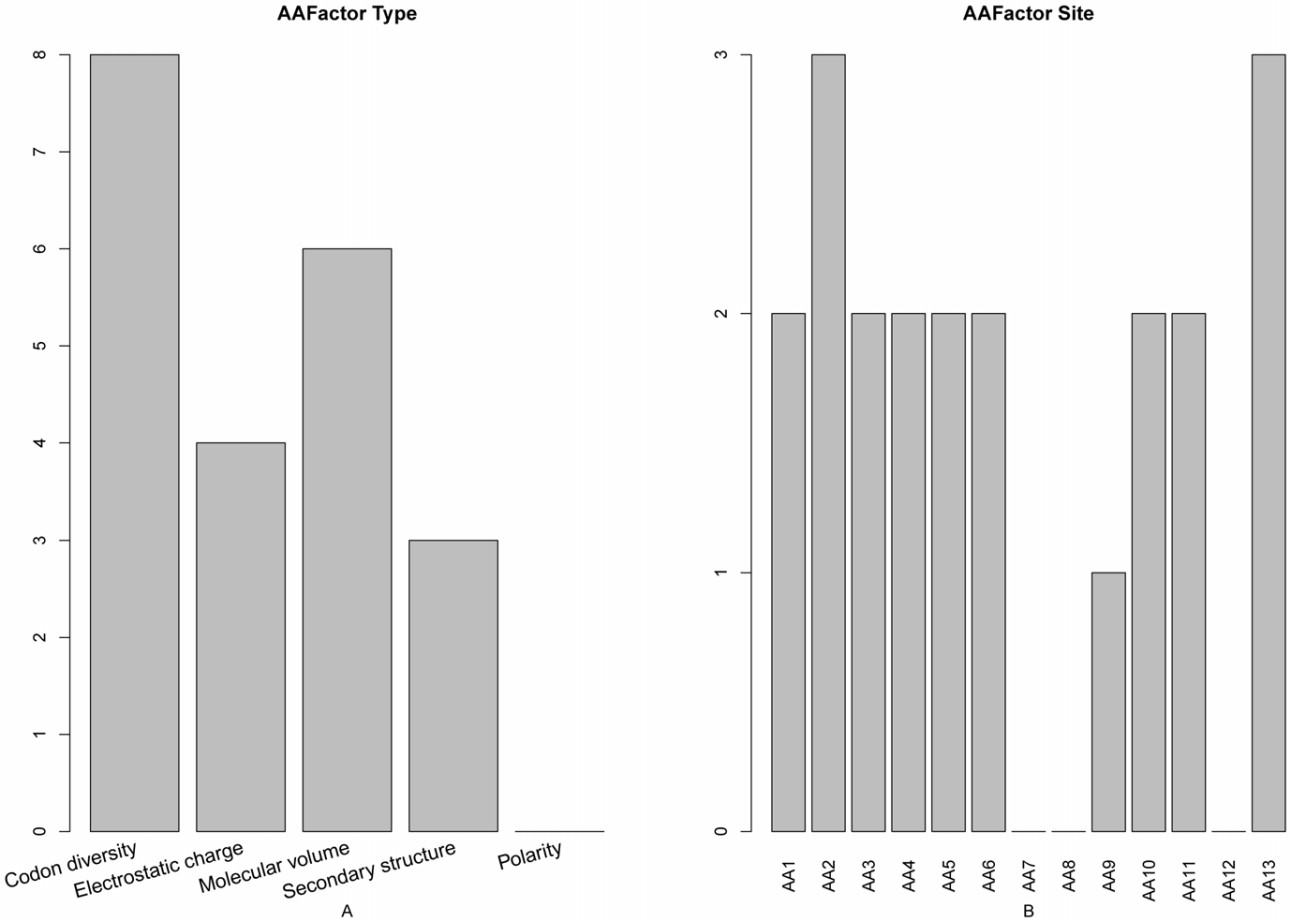
A 2-dimensional histogram to characterize the amino acid factor types in the final optimal features set. The impact on the domain prediction from (**A**) the five different amino acid types, and (**B**) each of the 13 subsites. See the text for further explanation.

Illustrated in **Fig. 4** are the impacts of different amino acid factors and their subsite locations to the protein domain prediction. It can be seen from **Fig. 4A** that the codon diversity was the most important feature to the protein domain site prediction, as supported by [98], [99]. Besides, it was reported that “codon harmonization” would put some non-preferred codons into the positions corresponding to the predicted protein domain boundaries [100]. Furthermore, the electrostatic charge has proved to be essential for the localization and activation of many proteins containing polycationic domains in their amino acid sequence [101]. Meanwhile, it has also been revealed that binding of oppositely charged proteins via electrostatic interactions can induce domain formation [102]. As shown in **Fig. 4B**, the amino acid residues at the subsite 2 and site 13 would have the highest impact to the protein domain sites prediction. Interestingly, the electrostatic feature at the subsite 13 had an index of 2 in our final optimal feature set, indicating that it was one of the most important features for the protein domain site prediction.

### 6. Disorder Analysis

Within the final optimal feature set, four of all the 13 disorder features were selected, indicating that the disorder feature might play a pivotal role in protein domain site prediction. Such four disorder features were from subsites 1, 5, 10 and 13. Particularly, the disorder feature of subsite 5 had the index of 1 in the final optimal feature set, suggesting that it was the most important feature in the protein domain site prediction. Also, the disorder feature of subsite 13 has an index of 9 in the final optimal feature site. These findings are fully consistent with the observations that the regions of substantial structural flexibility in a protein often correspond to domain boundaries where the structure is usually exposed and less constrained [39].

### 7. Solvent Accessibility Features Analysis

**Figure 5:**
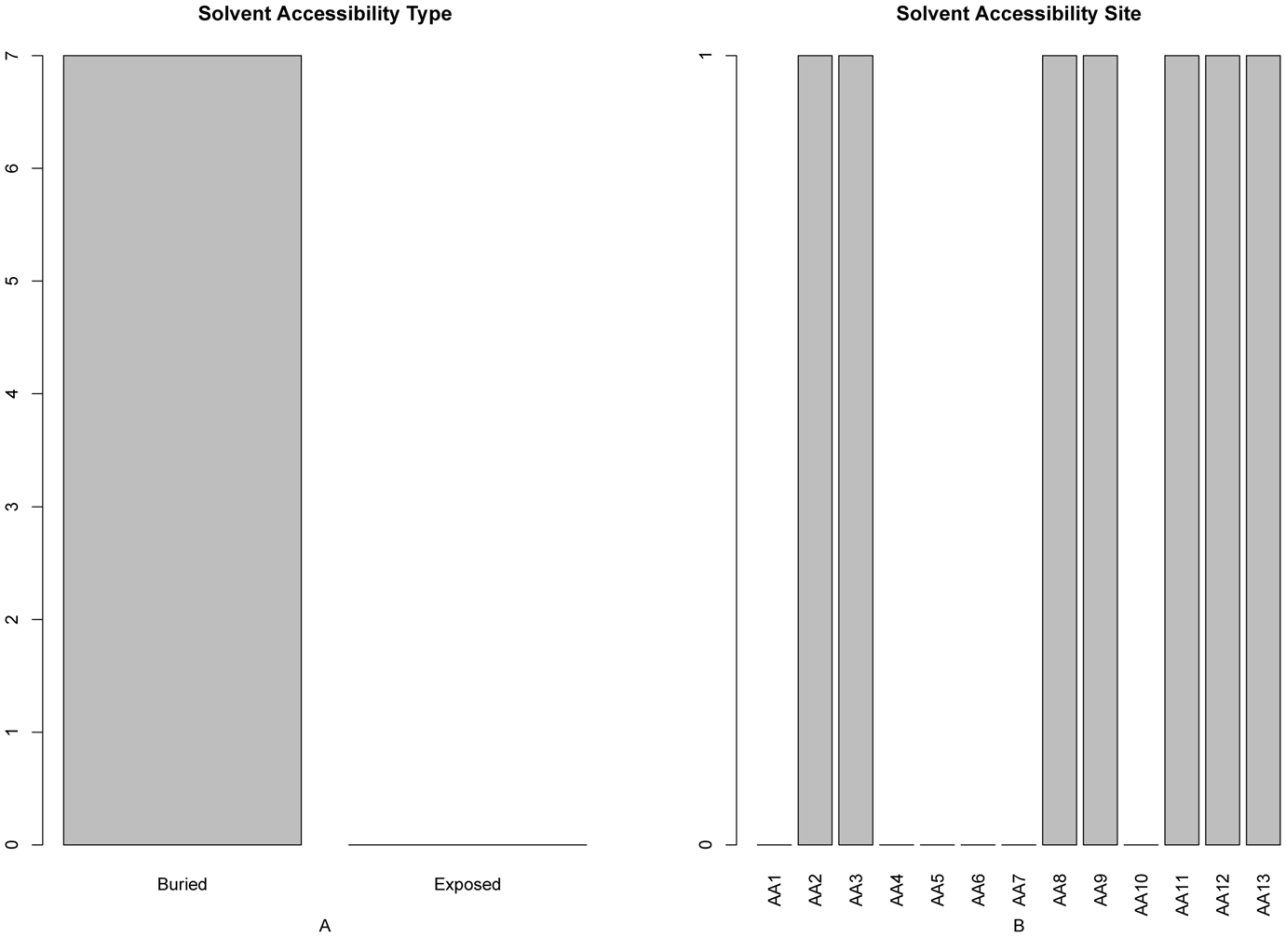
A 2-dimensional histogram to characterize the solvent accessibility types in the final optimal features set. The impact on the protein domain prediction from (**A**) the two different types of the solvent accessibility, and (**B**) each of the 13 subsites. See the text for further explanation.

Shown in **Fig. 5** are the solvent accessibility features in the optimal feature set. It can be seen from **Fig. 5A** that the number of buried solvent accessibility features was much more than that of the exposed, indicating that the protein domains were skewed toward the buried areas. Such findings are consistent with the report the buried protein regions can be accessible to water when they are in a free subunit or in one domain state and can form a complex or an aggregate with other subunits or domains [103]. Moreover, it can be seen from **Fig. 5B** that the solvent accessibility features at the subsites 2, 3, 8, 9, and 11–13 have relatively more impacts on the domain site prediction.

### 8. Secondary Structure Features Analysis

**Figure 6:**
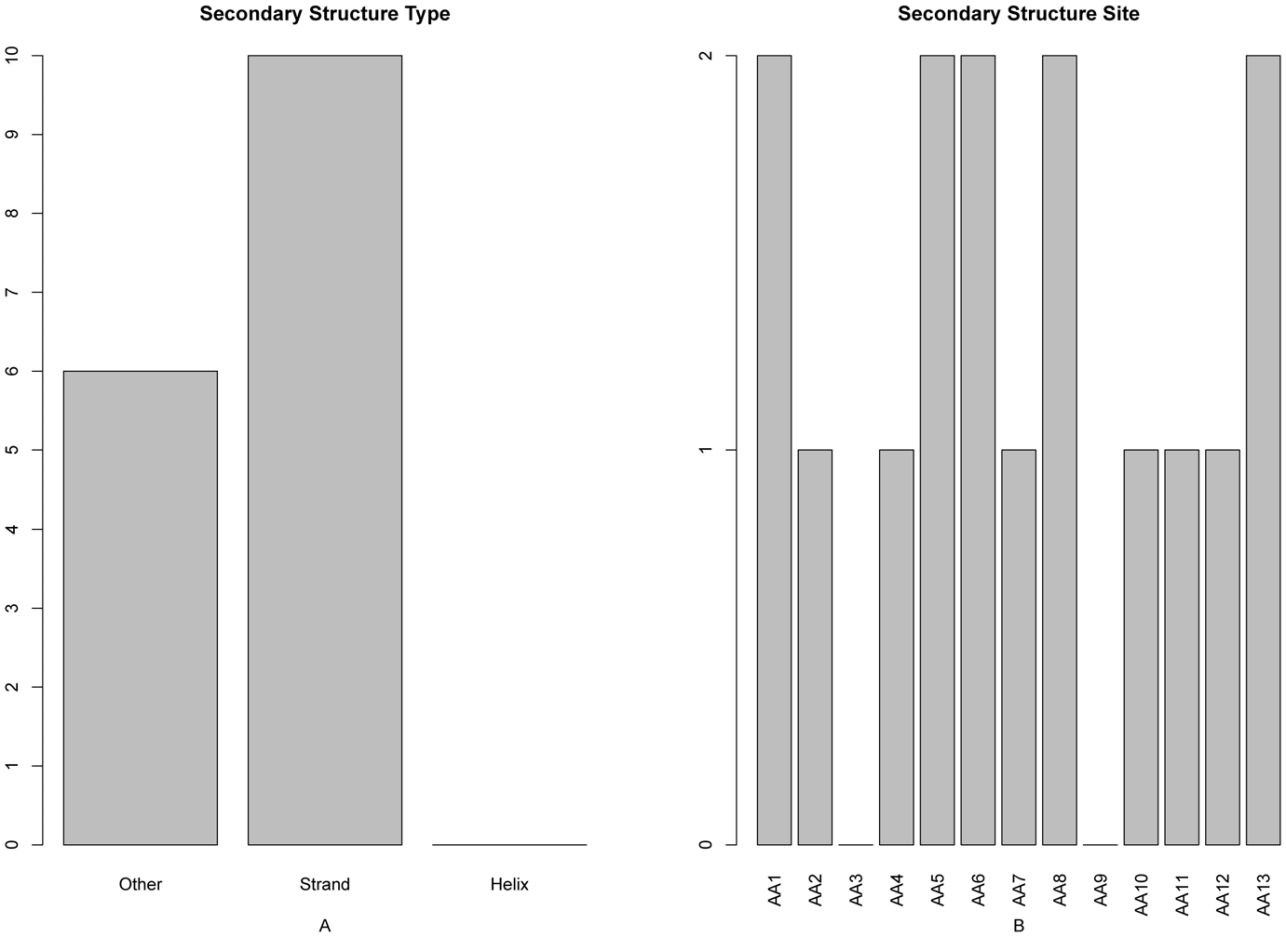
A 2-dimensional histogram to characterize the secondary structure types in the final optimal features set. The impact on the domain prediction from (**A**) the three different secondary structure types, and (**B**) each of the 13 subsites. See the text for further explanation. See the text for further explanation.

The feature and site-specific distribution of the secondary structure in the optimal feature set was given in **Fig. 6**, from which we can see that the features of “strand” and “other” did affect the domain site prediction (panel **A**), while the secondary structure features at subsites 1, 5, 6, 8 and 13 had relatively more impact on the domain site determination (panel **B**).

### 9. Scan the Entire Protein Sequence to Refine the Domain Region Prediction

**Figure 7:**
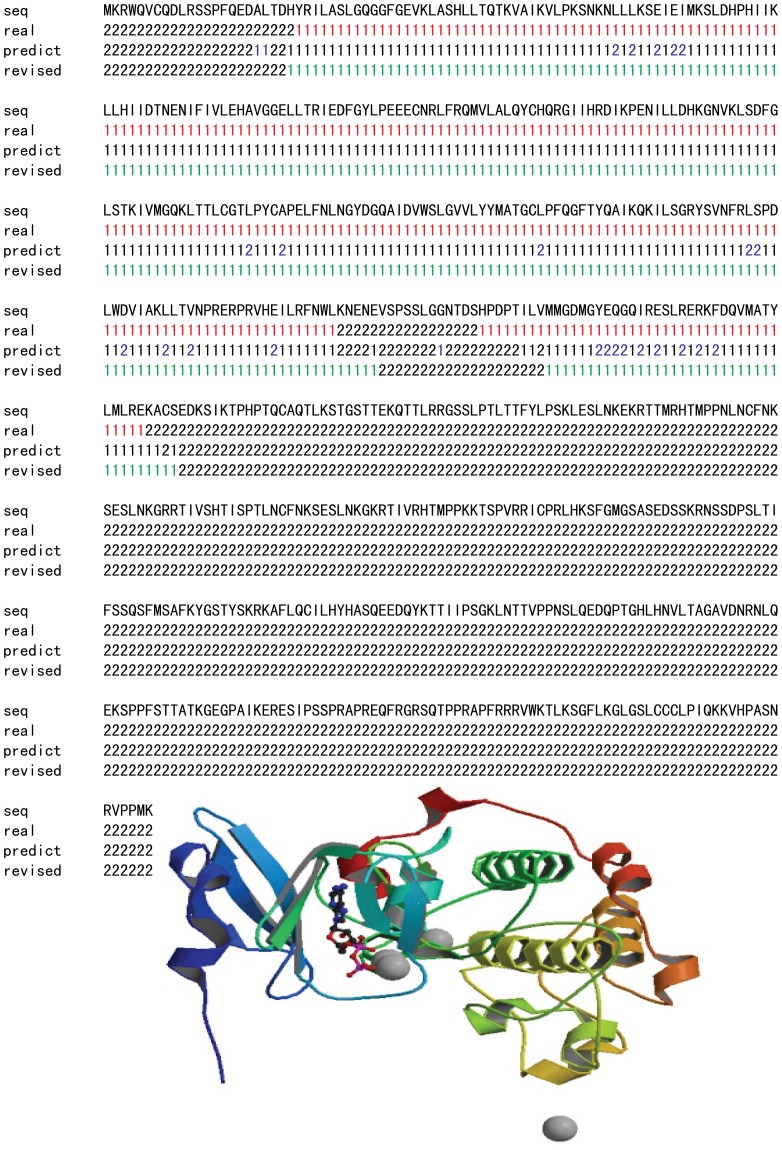
Illustration to show the predicted results obtained before and after applying the sequence-scanning refinement operation. A residue assigned to the domain region was coded with “1”; otherwise, “2”. The 3D structure of A1A5Q6 was retrieved from ModBase. See the text for further explanation.

As mentioned above, each of the amino acid residues in a protein sequence was identified whether it belonged to a domain region or non-domain region (cf. **Eq.2**). If a residue was identified as belonging to a domain region, it was coded with “1”; otherwise, “2”, as illustrated in **Fig. 7**. However, it is inevitable that some domain residues might be mispredicted as non-domain residues resulting in some short strand of “2” inserted in a long strand of “1” and vice versa. To filter out this kind of false positives and false negatives, a special scanning algorithm was developed to refine the entire predicted results according to the following criteria. (i) Any negative code “2” should be modified to a positive code “1” if it followed a strand of more than four continuous “1” codes but was followed by less than four continuous “2” codes. (ii) Any positive code “1” should be modified to a negative code “2” if it followed a strand of more than four continuous “2” codes but was followed by less than three continuous “1” codes. After such a scanning procedure, it can be seen from **Fig. 7** that many sporadic “2” codes in the long “1” regions have disappeared, and that many sporadic “1” codes in the long “2” regions have disappeared too. Meanwhile, the prediction quality was further improved as indicated in **Table 2**. Finally, the regions with the long continuous “1” codes thus obtained were assigned corresponding to the domain regions as indicated in Online Supporting Information S6.

**Table 2:** A comparison between the predicted results with and without the scanning refinement.

Window size	Scanning refinement^a^	Sensitivity^b^ 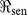	Specificity^b^ 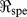	Accuracy^b^ 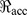	MCC^b^
13	No	0.578	0.794	0.728	0.367
	Yes	0.642	0.808	0.758	0.441
15	No	0.571	0.793	0.726	0.360
	Yes	0.634	0.806	0.754	0.431
17	No	0.574	0.793	0.726	0.362
	Yes	0.645	0.804	0.756	0.438

a See section 9 of Results and Discussion for more explanation about the scanning procedure.

b See Eq.14 for more explanation.

### 10. In Comparison with the Existing Methods

To evaluate our method, let us compare its performance with three existing methods in this area, including DoMpro [40], Globplot [49] and Domcut [48] based on the same testing dataset. Those methods such as FIEFDom [47] were not included because they were aimed at predicting domain boundaries rather than domains themselves. In other word, this kind of methods was based on such an assumption that nearly the whole protein was domain region except two or three domain boundaries. As a consequence, their sensitivity 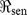 would be very close to 1, but the specificity 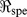 would be very low with quite poor overall success rates. The prediction result by the DoMpro [40] on a query protein sequence was formulated by a series of “N” and “T” codes to indicate that the corresponding residue being outside and inside the domain region, respectively. The predicted outcomes by the Globplot method [49] were the domain regions directly. As for the method Domcut [48], a score was assigned to each of the constituent residues in a query protein. The residues with a score below the cutoff threshold (default −0.09) were regarded as the inter-domain linker regions. For facilitating comparison, the results by all these methods on the same independent dataset 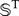
(cf. **Eq.1**) are also listed in **Table 3**, from which we can see that our method was about 58–70% higher than the other methods in specificity, 28–40% higher in accuracy, and 24–31% higher in MCC, but about 20% lower in sensitivity. These results indicate that the current method will play an important complementary role to the existing methods in identifying the domains of proteins.

**Table 3:** Comparison of the current method with the existing methods on the same testing dataset 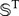 (cf. Eq.1).

Method	Sensitivity 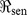	Specificity 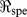	Accuracy 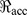	MCC
Our method	0.643	0.808	0.757	0.441
DoMpro [40]	0.924	0.182	0.406	0.138
Globplot [49]	0.868	0.325	0.485	0.199
Domcut [48] a	0.979	0.110	0.367	0.149
Domcut [48] b	0.856	0.325	0.482	0.186

a Using the default cutoff threshold of −0.09.

b Using the optimal cutoff threshold,

### 11. Useful Insights for Guiding Experiments or Being Validated by Experiments

The selected features at different sites may provide clues or insights for researchers to find or validate new protein domains, as can be viewed from the following four aspects. (**i**) PSSM feature. It was found through analyzing the PSSM conservation score that the mutations to amino acid residues N and D had the most impact on identifying the protein domain sites. Besides, the mutation to residues G and R also had more impacts than the other amino acids in this regard, fully consistent with the report [98] that D, G and R were over-represented in protein interaction domains, and the report [39] that amino acid G was instrumental in defining the core domain and interdomain regions of a protein. (**ii**) Codon diversity feature. It was revealed in this study that the codon diversity played pivotal role in identifying the protein domain sites, as evidenced by a series of experiments [98], [99], [100]. (**iii**) Electrostatic charge feature. It is interesting to note that electrostatic charge has proved to be essential for the localization and activation of many proteins containing polycationic domains in their amino acid sequence [101], and that binding of oppositely charged proteins via electrostatic interactions can induce domain formation [102]. All these observations are quite consistent with the findings in this study that the electrostatic feature of site 13 has an index of 2 in our final optimal feature set meaning that it is one of the most important features for the protein domain sites prediction. (**iv**) Disorder feature. It was found that in the final optimal feature set derived from this study, four of all the 13 disorder features were selected, and that disorder feature of site 5 had the index of 1, implying it was the most important feature to the protein domain site prediction. Interestingly, it has been reported that disorder regions often correspond to the domain boundaries [39]. Accordingly, the remainders in the optimal feature set are certainly worth being further investigated by future experiments.

It is anticipated that the strategy and approaches developed in this study may also be extended to investigate protein signal peptides (see, e.g., [60], [61], [104], [105]), B-cell epitopes [106], [107], HIV protease cleavage sites [108], [109], [110], [111], enzyme specificity [112], [113], among many other important topics in protein science and biomedicine.

## Supporting Information

Supporting Information S1 The UniProt ID codes for the proteins in the benchmark dataset: (A) training dataset and (B) testing dataset.
(XLSX)

Supporting Information S2 Detailed results obtained by the mRMR feature selection and analysis operation. It contains two sheets. The first one shows the 403 maximum relevance features of the size-13 window ranked according to their relevance with the classification of the samples. The second one shows the 403 mRMR features of the size-13 window ranked according to the redundancy and relevance criteria.
(XLSX)

Supporting Information S3 The results of the sensitivity (Sn), specificity (Sp), accuracy (Ac), and Matthews's correlation coefficient (MCC) versus the feature numbers. It contains three sheets. The first one shows the IFS results for the size-13 window, the second for the size-15 window, and the third for the size-17 window. The IFS curves were plotted based on the data in this file. See the text for more explanation.
(XLSX)

Supporting Information S4 The 360 features selected by the IFS procedure for the size-13 window. See the text for more explanation.
(XLSX)

Supporting Information S5 The 195 final optimal features selected by the incremental analysis procedure for the size-13 window. See the text for more explanation.
(XLSX)

Supporting Information S6 Domain region of the 1,299 proteins predicted by the method proposed in this paper.
(XLSX)

## References

[1] Chivian D, Kim DE, Malmstrom L, Bradley P, Robertson T (2003). Automated prediction of CASP-5 structures using the Robetta server.. Proteins.

[2] Ingolfsson H, Yona G (2008). Protein domain prediction.. Methods Mol Biol.

[3] Holland TA, Veretnik S, Shindyalov IN, Bourne PE (2006). Partitioning protein structures into domains: why is it so difficult?. J Mol Biol.

[4] Campbell ID, Downing AK (1994). Building protein structure and function from modular units.. Trends Biotechnol.

[5] Guerois R, Serrano L (2001). Protein design based on folding models.. Curr Opin Struct Biol.

[6] Nielsen PK, Yamada Y (2001). Identification of cell-binding sites on the Laminin alpha 5 N-terminal domain by site-directed mutagenesis.. J Biol Chem.

[7] Chou KC (2004). Review: Structural bioinformatics and its impact to biomedical science.. Current Medicinal Chemistry.

[8] Schnell JR, Chou JJ (2008). Structure and mechanism of the M2 proton channel of influenza A virus.. Nature.

[9] Wang J, Pielak RM, McClintock MA, Chou JJ (2009). Solution structure and functional analysis of the influenza B proton channel.. Nature Structural and Molecular Biology.

[10] Chou JJ, Li S, Klee CB, Bax A (2001). Solution structure of Ca2+-calmodulin reveals flexible hand-like properties of its domains.. Nature Structural Biology.

[11] Berardi MJ, Shih WM, Harrison SC, Chou JJ (2011). Mitochondrial uncoupling protein 2 structure determined by NMR molecular fragment searching.. Nature.

[12] Chou KC (2004). Insights from modelling the 3D structure of the extracellular domain of alpha7 nicotinic acetylcholine receptor.. Biochemical and Biophysical Research Communication.

[13] Chou KC (1995). The convergence-divergence duality in lectin domains of the selectin family and its implications.. FEBS Letters.

[14] Chou KC (2004). Modelling extracellular domains of GABA-A receptors: subtypes 1, 2, 3, and 5.. Biochemical and Biophysical Research Communications.

[15] Chou KC (2005). Modeling the tertiary structure of human cathepsin-E.. Biochem Biophys Res Commun.

[16] Chou KC (2001). Prediction of protein cellular attributes using pseudo amino acid composition.. PROTEINS: Structure, Function, and Genetics (Erratum: ibid, 2001, Vol44, 60).

[17] Chou KC (2011). Some remarks on protein attribute prediction and pseudo amino acid composition (50th Anniversary Year Review).. Journal of Theoretical Biology.

[18] Cai YD, Zhou GP, Chou KC (2003). Support vector machines for predicting membrane protein types by using functional domain composition.. Biophysical Journal.

[19] Xiao X, Wang P, Chou KC (2009). GPCR-CA: A cellular automaton image approach for predicting G-protein-coupled receptor functional classes.. Journal of Computational Chemistry.

[20] Xiao X, Wang P, Chou KC (2011). GPCR-2L: Predicting G protein-coupled receptors and their types by hybridizing two different modes of pseudo amino acid compositions.. Molecular Biosystems.

[21] Chou KC, Shen HB (2008). ProtIdent: A web server for identifying proteases and their types by fusing functional domain and sequential evolution information.. Biochem Biophys Res Comm.

[22] Xiao X, Wang P, Chou KC (2011). Quat-2L: a web-server for predicting protein quaternary structural attributes.. Molecular Diversity.

[23] Xiao X, Wang P, Chou KC (2009). Predicting protein quaternary structural attribute by hybridizing functional domain composition and pseudo amino acid composition.. Journal of Applied Crystallography.

[24] Chou KC, Cai YD (2004). Predicting protein structural class by functional domain composition.. Biochemical and Biophysical Research Communications (Corrigendum: ibid, 2005, Vol329, 1362).

[25] Wang K, Hu LL, Shi XH, Dong YS, Li HP (2012). PSCL: Predicting Protein Subcellular Localization Based on Optimal Functional Domains.. Protein & Peptide Letters.

[26] Chou KC, Shen HB (2010). A new method for predicting the subcellular localization of eukaryotic proteins with both single and multiple sites: Euk-mPLoc 2.0.. PLoS ONE.

[27] Chou KC, Shen HB (2010). Plant-mPLoc: A Top-Down Strategy to Augment the Power for Predicting Plant Protein Subcellular Localization.. PLoS ONE.

[28] Zhou GP (2011). The Structural Determinations of the Leucine Zipper Coiled-Coil Domains of the cGMP-Dependent Protein Kinase I alpha and its Interaction with the Myosin Binding Subunit of the Myosin Light Chains Phosphase.. Proteins & Peptide Letters.

[29] Zhou GP (2011). The disposition of the LZCC protein residues in wenxiang diagram provides new insights into the protein-protein interaction mechanism.. Journal of Theoretical Biology.

[30] Chen L, Feng KY, Cai YD, Chou KC, Li HP (2010). Predicting the network of substrate-enzyme-product triads by combining compound similarity and functional domain composition.. BMC Bioinformatics.

[31] Gewehr JE, Zimmer R (2006). SSEP-Domain: protein domain prediction by alignment of secondary structure elements and profiles.. Bioinformatics.

[32] von Ohsen N, Sommer I, Zimmer R, Lengauer T (2004). Arby: automatic protein structure prediction using profile-profile alignment and confidence measures.. Bioinformatics.

[33] Zhang Y, Liu B, Dong Q, Jin VX (2011). An improved profile-level domain linker propensity index for protein domain boundary prediction.. Protein & Peptide Letters.

[34] George RA, Heringa J (2002). SnapDRAGON: a method to delineate protein structural domains from sequence data.. J Mol Biol.

[35] Liu J, Rost B (2004). Sequence-based prediction of protein domains.. Nucleic Acids Res.

[36] Kim DE, Chivian D, Malmstrom L, Baker D (2005). Automated prediction of domain boundaries in CASP6 targets using Ginzu and RosettaDOM.. Proteins.

[37] Cheng J (2007). DOMAC: an accurate, hybrid protein domain prediction server.. Nucleic Acids Res.

[38] Shameer K, Pugalenthi G, Kandaswamy KK, Sowdhamini R (2011). 3dswap-pred: Prediction of 3D Domain Swapping from Protein Sequence Using Random Forest Approach.. Protein & Peptide Letters.

[39] Nagarajan N, Yona G (2004). Automatic prediction of protein domains from sequence information using a hybrid learning system.. Bioinformatics.

[40] Cheng J, Sweredoski M, Baldi P (2006). DOMpro: Protein Domain Prediction Using Profiles, Secondary Structure, Relative Solvent Accessibility, and Recursive Neural Networks.. Data Mining and Knowledge Discovery.

[41] Eickholt J, Deng X, Cheng J (2011). DoBo: Protein domain boundary prediction by integrating evolutionary signals and machine learning.. BMC Bioinformatics.

[42] Ebina T, Toh H, Kuroda Y (2011). DROP: an SVM domain linker predictor trained with optimal features selected by random forest.. Bioinformatics.

[43] Orengo CA, Michie AD, Jones S, Jones DT, Swindells MB (1997). CATH–a hierarchic classification of protein domain structures.. Structure.

[44] Murzin AG, Brenner SE, Hubbard T, Chothia C (1995). SCOP: a structural classification of proteins database for the investigation of sequences and structures.. J Mol Biol.

[45] Holm L, Sander C (1998). Dictionary of recurrent domains in protein structures.. Proteins.

[46] Walsh I, Martin AJ, Mooney C, Rubagotti E, Vullo A (2009). Ab initio and homology based prediction of protein domains by recursive neural networks.. BMC Bioinformatics.

[47] Bondugula R, Lee MS, Wallqvist A (2009). FIEFDom: a transparent domain boundary recognition system using a fuzzy mean operator.. Nucleic Acids Res.

[48] Suyama M, Ohara O (2003). DomCut: prediction of inter-domain linker regions in amino acid sequences.. Bioinformatics.

[49] Linding R, Russell RB, Neduva V, Gibson TJ (2003). GlobPlot: Exploring protein sequences for globularity and disorder.. Nucleic Acids Res.

[50] Wu ZC, Xiao X, Chou KC (2011). iLoc-Plant: a multi-label classifier for predicting the subcellular localization of plant proteins with both single and multiple sites.. Molecular BioSystems.

[51] Xiao X, Wu ZC, Chou KC (2011). A multi-label classifier for predicting the subcellular localization of gram-negative bacterial proteins with both single and multiple sites.. PLoS ONE.

[52] Lin WZ, Fang JA, Xiao X, Chou KC (2011). iDNA-Prot: Identification of DNA Binding Proteins Using Random Forest with Grey Model.. PLoS ONE.

[53] Wang P, Xiao X, Chou KC (2011). NR-2L: A Two-Level Predictor for Identifying Nuclear Receptor Subfamilies Based on Sequence-Derived Features.. PLoS ONE.

[54] Chou KC, Wu ZC, Xiao X (2012). iLoc-Hum: Using accumulation-label scale to predict subcellular locations of human proteins with both single and multiple sites.. Molecular Biosystems.

[55] Xiao X, Wu ZC, Chou KC (2011). iLoc-Virus: A multi-label learning classifier for identifying the subcellular localization of virus proteins with both single and multiple sites.. Journal of Theoretical Biology.

[56] Wu ZC, Xiao X, Chou KC (2012). iLoc-Gpos: A Multi-Layer Classifier for Predicting the Subcellular Localization of Singleplex and Multiplex Gram-Positive Bacterial Proteins.. Protein & Peptide Letters.

[57] Apweiler R, Martin MJ, O'Donovan C, Magrane M, Alam-Faruque Y (2010). The Universal Protein Resource (UniProt) in 2010.. Nucleic Acids Research.

[58] Chou KC, Shen HB (2007). Review: Recent progresses in protein subcellular location prediction.. Analytical Biochemistry.

[59] Li W, Godzik A (2006). Cd-hit: a fast program for clustering and comparing large sets of protein or nucleotide sequences.. Bioinformatics.

[60] Chou KC (2002). Review: Prediction of protein signal sequences.. Current Protein and Peptide Science.

[61] Chou KC, Shen HB (2007). Signal-CF: a subsite-coupled and window-fusing approach for predicting signal peptides.. Biochem Biophys Res Comm.

[62] Schaffer AA, Aravind L, Madden TL, Shavirin S, Spouge JL (2001). Improving the accuracy of PSI-BLAST protein database searches with composition-based statistics and other refinements.. Nucleic Acids Res.

[63] Altschul SF, Madden TL, Schaffer AA, Zhang J, Zhang Z (1997). Gapped BLAST and PSI-BLAST: a new generation of protein database search programs.. Nucleic Acids Res.

[64] Chou KC, Shen HB (2007). MemType-2L: A Web server for predicting membrane proteins and their types by incorporating evolution information through Pse-PSSM.. Biochem Biophys Res Comm.

[65] Hayat M, Khan A (2012). MemHyb: Predicting membrane protein types by hybridizing SAAC and PSSM.. J ournal of Theoretical Biology.

[66] Li D, Jiang Z, Yu W, Du L (2010). Predicting Caspase Substrate Cleavage Sites Based on a Hybrid SVM-PSSM Method.. Protein and Peptide Letters.

[67] Mundra P, Kumar M, Kumar KK, Jayaraman VK, Kulkarni BD (2007). Using pseudo amino acid composition to predict protein subnuclear localization: Approached with PSSM.. Pattern Recognition Letters.

[68] Shen HB, Chou KC (2007). Nuc-PLoc: A new web-server for predicting protein subnuclear localization by fusing PseAA composition and PsePSSM.. Protein Engineering, Design & Selection.

[69] Chou KC, Wu ZC, Xiao X (2011). iLoc-Euk: A Multi-Label Classifier for Predicting the Subcellular Localization of Singleplex and Multiplex Eukaryotic Proteins.. PLoS One.

[70] Kawashima S, Kanehisa M (2000). AAindex: amino acid index database.. Nucleic Acids Res.

[71] Atchley WR, Zhao J, Fernandes AD, Druke T (2005). Solving the protein sequence metric problem.. Proc Natl Acad Sci U S A.

[72] Wright PE, Dyson HJ (1999). Intrinsically unstructured proteins: re-assessing the protein structure-function paradigm.. Journal of Molecular Biology.

[73] Dunker AK, Brown CJ, Lawson JD, Iakoucheva LM, Obradovic Z (2002). Intrinsic disorder and protein function.. Biochemistry.

[74] Yoon MK, Venkatachalam V, Huang A, Choi BS, Stultz CM (2009). Residual structure within the disordered C-terminal segment of p21(Waf1/Cip1/Sdi1) and its implications for molecular recognition.. Protein Sci.

[75] Liu J, Tan H, Rost B (2002). Loopy proteins appear conserved in evolution.. Journal of Molecular Biology.

[76] Tompa P (2002). Intrinsically unstructured proteins.. Trends in Biochemical Sciences.

[77] Peng K, Radivojac P, Vucetic S, Dunker AK, Obradovic Z (2006). Length-dependent prediction of protein intrinsic disorder.. BMC Bioinformatics.

[78] Cheng J, Randall AZ, Sweredoski MJ, Baldi P (2005). SCRATCH: a protein structure and structural feature prediction server.. Nucleic Acids Research.

[79] Peng H, Long F, Ding C (2005). Feature selection based on mutual information: criteria of max-dependency, max-relevance, and min-redundancy.. IEEE Trans Pattern Anal Mach Intell.

[80] Kandaswamy KK, Chou KC, Martinetz T, Moller S, Suganthan PN (2011). AFP-Pred: A random forest approach for predicting antifreeze proteins from sequence-derived properties.. Journal of Theoretical Biology.

[81] Pugalenthi G, Kandaswamy KK, Chou KC, Vivekanandan S, Kolatkar P (2012). RSARF: Prediction of Residue Solvent Accessibility from Protein Sequence Using Random Forest Method.. Protein & Peptide Letters.

[82] Jia SC, Hu XZ (2011). Using Random Forest Algorithm to Predict beta-Hairpin Motifs.. Protein and Peptide Letters.

[83] Qiu Z, Wang X (2011). Improved Prediction of Protein Ligand-Binding Sites Using Random Forests.. Protein & Peptide Letters.

[84] Breiman L (2001). Random forests.. Machine learning.

[85] Witten IH, Frank E (2005).

[86] Chou KC, Zhang CT (1995). Review: Prediction of protein structural classes.. Critical Reviews in Biochemistry and Molecular Biology.

[87] Esmaeili M, Mohabatkar H, Mohsenzadeh S (2010). Using the concept of Chou's pseudo amino acid composition for risk type prediction of human papillomaviruses.. Journal of Theoretical Biology.

[88] Mohabatkar H, Mohammad Beigi M, Esmaeili A (2011). Prediction of GABA(A) receptor proteins using the concept of Chou's pseudo-amino acid composition and support vector machine.. Journal of Theoretical Biology.

[89] Lin J, Wang Y (2011). Using a novel AdaBoost algorithm and Chou's pseudo amino acid composition for predicting protein subcellular localization.. Protein & Peptide Letters.

[90] Gu Q, Ding YS, Zhang TL (2010). Prediction of G-Protein-Coupled Receptor Classes in Low Homology Using Chou's Pseudo Amino Acid Composition with Approximate Entropy and Hydrophobicity Patterns.. Protein & Peptide Letters.

[91] Xiao X, Wang P, Chou KC (2012). iNR-PhysChem: A Sequence-Based Predictor for Identifying Nuclear Receptors and Their Subfamilies via Physical-Chemical Property Matrix.. PLoS ONE.

[92] Li YX, Shao YH, Jing L, Deng NY (2011). An efficient support vector machine approach for identifying protein s-nitrosylation sites.. Protein and Peptide Letters.

[93] Qiu JD, Huang JH, Shi SP, Liang RP (2010). Using the concept of Chou's pseudo amino acid composition to predict enzyme family classes: an approach with support vector machine based on discrete wavelet transform.. Protein & Peptide Letters.

[94] Zhao W, Wang X, Deng R, Wang J, Zhou H (2011). Discrimination of Thermostable and Thermophilic Lipases using Support Vector Machines.. Protein & Peptide Letters.

[95] Huang T, Chen L, Cai YD, Chou KC (2011). Classification and analysis of regulatory pathways using graph property, biochemical and physicochemical property, and functional property.. PLoS ONE.

[96] He Z, Zhang J, Shi XH, Hu LL, Kong X (2010). Predicting drug-target interaction networks based on functional groups and biological features.. PLoS ONE.

[97] Li BQ, Hu LL, Niu S, Cai YD, Chou KC (2012). Predict and analyze S-nitrosylation modification sites with the mRMR and IFS approaches.. J Proteomics.

[98] Roy S, Martinez D, Platero H, Lane T, Werner-Washburne M (2009). Exploiting amino acid composition for predicting protein-protein interactions.. PLoS One.

[99] Moses AM, Durbin R (2009). Inferring selection on amino acid preference in protein domains.. Mol Biol Evol.

[100] Angov E, Hillier CJ, Kincaid RL, Lyon JA (2008). Heterologous protein expression is enhanced by harmonizing the codon usage frequencies of the target gene with those of the expression host.. PLoS One.

[101] Goldenberg NM, Steinberg BE (2010). Surface charge: a key determinant of protein localization and function.. Cancer Res.

[102] Mbamala EC, Ben-Shaul A, May S (2005). Domain formation induced by the adsorption of charged proteins on mixed lipid membranes.. Biophys J.

[103] Gong S, Park C, Choi H, Ko J, Jang I (2005). A protein domain interaction interface database: InterPare.. BMC Bioinformatics.

[104] Li YD, Zhou Z, Lv LX, Hou XP, Li YQ (2009). New approach to achieve high-level secretory expression of heterologous proteins by using Tat signal peptide.. Protein & Peptide Letters.

[105] Reynolds SM, Kall L, Riffle ME, Bilmes JA, Noble WS (2008). Transmembrane topology and signal peptide prediction using dynamic bayesian networks.. PLoS Comput Biol.

[106] Saffari B, Mohabatkar H, Mohsenzadeh S (2008). T and B-cell Epitopes Prediction of Iranian Saffron (Crocus sativus) Profilin by Bioinformatics Tools.. Protein Pept Lett.

[107] Chen J, Liu H, Yang J, Chou KC (2007). Prediction of linear B-cell epitopes using amino acid pair antigenicity scale.. Amino Acids.

[108] Chou KC (1993). A vectorized sequence-coupling model for predicting HIV protease cleavage sites in proteins.. Journal of Biological Chemistry.

[109] Poorman RA, Tomasselli AG, Heinrikson RL, Kezdy FJ (1991). A cumulative specificity model for proteases from human immunodeficiency virus types 1 and 2, inferred from statistical analysis of an extended substrate data base.. Journal of Biological Chemistry.

[110] Chou KC (1996). Review: Prediction of HIV protease cleavage sites in proteins.. Analytical Biochemistry.

[111] Shen HB, Chou KC (2008). HIVcleave: a web-server for predicting HIV protease cleavage sites in proteins.. Analytical Biochemistry.

[112] Chou KC (1995). A sequence-coupled vector-projection model for predicting the specificity of GalNAc-transferase.. Protein Science.

[113] Elhammer AP, Poorman RA, Brown E, Maggiora LL, Hoogerheide JG (1993). The specificity of UDP-GalNAc:polypeptide N-acetylgalactosaminyltransferase as inferred from a database of in vivo substrates and from the in vitro glycosylation of proteins and peptides.. Journal of Biological Chemistry.

